# Case Report: Probable manic episode induced by PEG-IFN *α*-2b in a chronic hepatitis B patient—multidisciplinary management insights

**DOI:** 10.3389/fmed.2026.1849394

**Published:** 2026-06-02

**Authors:** Xiao-Cui Wu, Hui Zhou, Lan-Tian Pang, Cui-Lan Tang

**Affiliations:** Department of Infectious Diseases, The Second Affiliated Hospital, Zhejiang University School of Medicine, Hangzhou, Zhejiang, China

**Keywords:** adverse drug reaction, chronic hepatitis B, functional cure, manic episode, pegylated interferon alfa-2b

## Abstract

**Background:**

Pegylated interferon alfa-2b (PEG-IFN *α*-2b) is a first-line treatment for achieving functional cure in chronic hepatitis B (CHB). Although neuropsychiatric adverse events such as depression and sleep disturbances are well-documented with PEG-IFN *α*-2b treatment, manic episodes are exceptionally rare, especially in CHB patients with no prior psychiatric risk factors.

**Case presentation:**

A middle-aged male CHB patient with no personal or family history of mental disorders developed a manic episode at week 33 of PEG-IFN *α*-2b therapy. He was initially managed at a psychiatric hospital, then transferred to our hepatology department due to interferon-related thrombocytopenia. A multidisciplinary team (MDT) comprising hepatologists, psychiatrists, neurologists, and pharmacists conducted an etiological investigation, which confirmed a high likelihood of PEG-IFN *α*-2b-induced manic episode.

**Management and outcomes:**

Discontinuing PEG-IFN α-2b and initiating targeted antipsychotic treatment led to rapid resolution of psychiatric symptoms and recovery of platelet count. At the 2-week follow-up, the patient’s mood was stable with a Young Mania Rating Scale score of 4, and his platelet count recovered to 334 × 10^9^/L by week 36 of follow-up.

**Conclusion:**

Manic episodes can occur during PEG-IFN *α*-2b therapy for CHB even in patients without psychiatric risk factors. Clinicians should remain vigilant for this rare adverse event and implement routine mental health monitoring. MDT collaboration is critical for accurate etiological diagnosis and optimal management, ensuring a balance between pursuing functional cure and safeguarding treatment safety.

## Introduction

Chronic hepatitis B (CHB) remains a major global public health challenge, affecting approximately 254 million people worldwide (1). Achieving functional cure, defined as sustained loss of hepatitis B surface antigen (HBsAg) with undetectable serum HBV DNA 24 weeks after treatment, is critical for improving long-term patient outcomes and reducing HBV-related complications like cirrhosis and hepatocellular carcinoma (2, 3). Unlike conventional interferon alfa, which requires frequent dosing, pegylated interferon alfa-2b (PEG-IFN *α*-2b) extends the drug’s half-life to enable weekly subcutaneous administration, and its dual antiviral and immunomodulatory properties enhance functional cure rates, rendering it a first-line therapeutic option for eligible patients with CHB (4).

PEG-IFN is associated with a range of adverse events, including flu-like symptoms, myelosuppression, and transient transaminase elevation. Neuropsychiatric manifestations are also common, with depression and sleep disturbances being the most prevalent; these are usually mild and reversible (5). In contrast, manic episodes induced by PEG-IFN *α*-2b are extremely rare (6). Among patients with viral hepatitis, most reported cases occur in those with chronic hepatitis C virus (HCV) infection (7–9). Additionally, patients with pre-existing psychiatric disorders are more prone to developing neuropsychiatric adverse effects during interferon therapy (10). Consistent with this, the “Update on the Treatment Navigation for Functional Cure of Chronic Hepatitis B: Expert Consensus 2.0” specifies that patients with active psychiatric disorders or a history of suicidal ideation are contraindicated for PEG-IFN-based curative therapy. Given the rarity of PEG-IFN *α*-2b-induced manic episodes, particularly in patients without psychiatric risk factors, we present this case to emphasize the necessity of clinical vigilance and the importance of multidisciplinary collaboration in the diagnosis and management of this rare adverse event.

## Case presentation

### Chief complaint

Positive hepatitis B surface antigen (HBsAg) for 18 years and mental abnormalities for 1 week.

### History of the present illness

A 46-year-old Han Chinese male was diagnosed with CHB 18 years earlier (HBsAg-positive). He initially received oral lamivudine for antiviral therapy, which was switched to tenofovir disoproxil fumarate (TDF) 8 years ago due to lamivudine resistance.

On February 26, 2025, the patient started PEG-IFN *α*-2b therapy (180 μg/0.5 mL subcutaneously once weekly) to pursue functional cure. Baseline evaluations showed HBsAg 577.98 IU/mL, undetectable HBV DNA, normal liver function, thyroid function, and antinuclear antibody (ANA), as well as a liver stiffness of 5.66 kPa.

During follow-up, HBsAg levels gradually decreased to 487.88 IU/mL at week 12 and 134.17 IU/mL at week 24, with persistently undetectable HBV DNA. Mild leukopenia and thrombocytopenia were noted during treatment, which resolved with symptomatic management without interrupting PEG-IFN *α*-2b therapy.

At week 33 of PEG-IFN α-2b therapy, the patient developed acute emotional lability and impulsive behaviors. He was admitted to a psychiatric specialty hospital, where a manic episode was confirmed with a Young Mania Rating Scale (YMRS) score of 37. He received symptomatic treatment (including diazepam for agitation, quetiapine and valproate for mood stabilization, and amisulpride for manic symptom control), which partially relieved his psychiatric symptoms. However, a recheck of the complete blood count (CBC) showed a platelet count of 49 × 10^9^/L (reference range: 100–300 × 10^9^/L), prompting transfer to our department for further management of CHB-related complications.

### Past medical history

He had a 1-year history of hypertension, well-controlled with oral amlodipine besylate (5 mg once daily).

### Personal and family history

The patient denied smoking, alcohol consumption, or illicit drug use. He had no personal or family history of psychiatric disorders, infectious diseases, genetic diseases, or malignancies.

### Physical examination

The patient was conscious and alert, with mild mental fatigue, stable mood, and slurred speech. No jaundice, hepatosplenomegaly, or edema was observed. Cardiac rhythm was regular, and bilateral breath sounds were coarse without rales. Neurological examination yielded no abnormal findings.

### Diagnostic investigations

CBC revealed a white blood cell count of 2.1 × 10^9^/L, a hemoglobin level of 112 g/L, and a platelet count of 42 × 10^9^/L. The HBsAg level was 577.98 IU/mL, and HBV DNA was undetectable. Liver and kidney function, myocardial enzymes, electrolytes, thyroid hormones, ANA, autoimmune liver disease markers, and blood ammonia were all within normal ranges. Cytomegalovirus/Epstein–Barr virus serology and nucleic acid tests were negative.

### Imaging findings

No obvious abnormalities were found on the B-ultrasound of the hepatobiliary, pancreatic, and splenic organs. Cranial magnetic resonance imaging (fluid-attenuated inversion recovery/diffusion-weighted imaging sequences) showed no abnormal lesions.

## Multidisciplinary collaboration

An MDT consultation involving hepatologists, psychiatrists, neurologists, and pharmacists was organized. Key findings included: (1) Acute onset of manic symptoms and thrombocytopenia temporally associated with long-term PEG-IFN *α*-2b exposure; (2) No personal or family history of psychiatric disorders, and no concurrent medications linked to mania; (3) Exclusion of organic brain lesions, hepatic encephalopathy, and other infectious or metabolic causes; (4) Partial symptom improvement with antipsychotic treatment prior to transfer. Based on these findings, a diagnosis of PEG-IFN *α*-2b-induced secondary manic episode was made. The Naranjo Adverse Drug Reaction Probability Scale score was 7, indicating “probable” causality.

## Final diagnosis

PEG-IFN *α*-2b-induced manic episode; chronic hepatitis B; hypertension.

## Treatment and outcome

PEG-IFN α-2b was immediately discontinued upon admission. The treatment regimen included: (1) Antiviral therapy: Oral TDF 0.3 g once daily; (2) Hypertension control: Oral amlodipine besylate 5 mg once daily; (3) Hematological support: Oral leucogen (20 mg three times daily), Shengxuexiaoban Capsule (1.8 g three times daily), and a single intravenous dose of recombinant human thrombopoietin (15,000 units); (4) Psychiatric intervention: Oral quetiapine fumarate (0.1 g at 11:00 and 0.2 g at 17:00 daily), valproate (0.2 g twice daily), and paliperidone extended-release tablets (3 mg daily).

Valproate was discontinued on hospital day 5. The patient was discharged on hospital day 6, continuing oral quetiapine and paliperidone extended-release tablets. At the 2-week follow-up at the psychiatry clinic, his mood was stable with a YMRS score of 4, clear expression, and no subjective discomfort. Paliperidone extended-release tablets were discontinued, and he remained on oral quetiapine fumarate monotherapy. At week 36 of follow-up, his platelet count had recovered to 334 × 10^9^/L.

## Discussion

PEG-IFN *α*-2b is widely used in CHB treatment due to its potential to achieve functional cure (11). While neuropsychiatric adverse events are known to occur with interferon therapy, manic episodes are exceptionally rare (12, 13). Most reported cases occur in patients with HCV infection. However, interferon-induced manic episodes have been rarely reported in patients with CHB, making this case clinically notable.

Notably, IFN-induced adverse events typically occur within days to weeks of initiation and are mild (14). In this case, manic symptoms emerged at week 33, consistent with literature suggesting that late-onset IFN-induced neuropsychiatric manifestations may be more severe (15). Patient records indicated that prior to the initiation of PEG-IFN *α*-2b therapy, the patient had no history of psychiatric disorders, drug or alcohol abuse, or a family history of psychiatric disorders. Additionally, the medical records, along with confirmations from the patient’s family members and attending physicians, verified that the patient was not receiving any concurrent medications. After admission to the psychiatric hospital, PEG-IFN *α*-2b therapy was discontinued due to the patient’s prominent psychotic symptoms. Symptomatic relief was achieved after the administration of antipsychotic medications.

During this period, liver function, renal function, myocardial enzymes, electrolytes, thyroid hormones, antinuclear antibodies (ANA), and autoimmune liver disease markers were all within the normal range. No abnormal lesions were detected on brain magnetic resonance imaging (MRI). To further rule out the possibility of hepatic encephalopathy, blood ammonia levels were measured, and the results were also within the normal range. Of note, CBC revealed a white blood cell count of 2.1 × 10^9^/L and a platelet count of 42 × 10^9^/L. It is known that PEG-IFN can cause hematological adverse effects. The concurrent thrombocytopenia observed in this case also resolved after drug discontinuation, which further supports the diagnosis that this event was a PEG-IFN *α*-2b related adverse reaction caused by multiple factors.

The pathogenesis of IFN-induced mania remains unclear; potential mechanisms include neurotransmitter imbalance (e.g., serotonin depletion, dopamine dysregulation) (16, 17), neuroinflammation (18, 19), and neuroendocrine disturbances (e.g., hypothalamic–pituitary–adrenal axis hyperactivity) (20, 21). However, the exact mechanism in this patient requires further investigation.

This case highlights two key clinical points: First, clinicians should maintain heightened vigilance for manic episodes during PEG-IFN *α*-2b therapy for CHB, even in patients without psychiatric risk factors. Routine mental health screening and patient/family education on potential neuropsychiatric symptoms are essential for early detection. Second, MDT collaboration is vital for accurate etiological diagnosis and optimal management. Given the complexity of overlapping psychiatric symptoms and CHB-related complications (e.g., thrombocytopenia), interdisciplinary input from hepatologists, psychiatrists, neurologists, and pharmacists ensures timely identification of the cause, appropriate treatment adjustments (e.g., PEG-IFN discontinuation), and targeted interventions—ultimately improving patient outcomes. In the diagnosis and treatment of this case, the hepatologist led the management of CHB and its complications (e.g., thrombocytopenia). The psychiatrist confirmed the diagnosis of mania and guided antipsychotic therapy. The neurologist helped rule out organic central nervous system disorders. The pharmacist contributed by reviewing the medication history, assessing the temporal relationship with PEG-IFN *α*-2b, and evaluating other drug-related causes, which was essential for the Naranjo scale assessment.

## Limitations

This report is limited by its single-case design, which prevents determining the incidence or specific risk factors (e.g., age, dosage) of PEG-IFN *α*-2b-induced mania in CHB patients. Future multicenter retrospective studies or prospective cohorts are needed to further characterize this rare adverse event.

## Conclusion

PEG-IFN *α*-2b-induced manic episodes can occur in CHB patients without pre-existing psychiatric risk factors. Clinicians should integrate routine mental health monitoring into PEG-IFN therapy and remain alert to this rare but serious adverse event. Multidisciplinary collaboration is indispensable for accurate diagnosis and effective management, balancing the pursuit of functional cure with treatment safety.

## Data Availability

The original contributions presented in the study are included in the article/supplementary material, further inquiries can be directed to the corresponding author.
